# Development of ERK Activity Sensor, an *in vitro*, FRET-based sensor of Extracellular Regulated Kinase activity

**DOI:** 10.1186/1472-6769-5-1

**Published:** 2005-07-05

**Authors:** Harry M Green, José Alberola-Ila

**Affiliations:** 1 Division of Biology, California Institute of Technology, M/C 147-75, 1200 E. California Blvd, Pasadena, CA 91125, USA

## Abstract

**Background:**

Study of ERK activation has thus far relied on biochemical assays that are limited to the use of phospho-specific antibodies and radioactivity *in vitro*, and analysis of whole cell populations *in vivo*. As with many systems, fluorescence resonance energy transfer (FRET) can be utilized to make highly sensitive detectors of molecular activity. Here we introduce FRET-based ERK Activity Sensors, which utilize variants of Enhanced Green Fluorescent Protein fused by an ERK-specific peptide linker to detect ERK2 activity.

**Results:**

ERK Activity Sensors display varying changes in FRET upon phosphorylation by active ERK2 *in vitro *depending on the composition of ERK-specific peptide linker sequences derived from known *in vivo *ERK targets, Ets1 and Elk1. Analysis of point mutations reveals specific residues involved in ERK binding and phosphorylation of ERK Activity Sensor 3. ERK2 also shows high *in vitro *specificity for these sensors over two other major MAP Kinases, p38 and pSAPK/JNK.

**Conclusion:**

EAS's are a convenient, non-radioactive alternative to study ERK dynamics *in vitro*. They can be utilized to study ERK activity in real-time. This new technology can be applied to studying ERK kinetics *in vitro*, analysis of ERK activity in whole cell extracts, and high-throughput screening technologies.

## Background

Traditional methods for studying signal transduction cascades have been based solely on biochemical analysis of whole cell populations and homogenized tissues (e.g. radioassays, western blots, etc.). In addition, *in vitro *studies have required the use of radioactive isotopes for biochemical characterization of kinases. These methods are time consuming, produce large quantities of radioactive waste, and do not allow for the study of real-time kinase dynamics.

Recently, sensors for studying signal molecules *in vitro*, as well as cascade dynamics in single cells, have been developed utilizing fluorescent proteins and the phenomenon of fluorescence resonance energy transfer (FRET). FRET is a phenomenon by which energy is transferred from one fluorescent molecule to another by way of dipole-dipole interactions during excitation of the donor molecule. FRET efficiency is given by the equation:



where R is the donor-acceptor radius and R_o _is the radius at which FRET efficiency is 50% (Förster radius). Small changes in R (1–2Å) and small orientation changes between the donor and acceptor fluorophores can dramatically affect the efficiency of FRET, making very small changes in structure easily detectable. The limitation, however, is that FRET is effective only between 10–100Å, and therefore the donor and acceptor must be maintained in close proximity. Genetically encoded fluorescent proteins fused by linker peptide sequences have been utilized to address proximity limitations, as well as facilitate the delivery of the sensor into live cells.

Some of the earliest work done with these sensors includes genetically encoded sensors of caspase activity, in which caspase-3 and caspase-8 sensitive linker peptides were fused between the different variants of Enhanced Green Fluorescent Protein (EGFP) [1]. These studies allowed the characterization of apoptosis in single living cells with spatio-temporal resolution. Other types of FRET signaling sensors have since evolved, including sensors for calcium signaling [2], receptor tyrosine kinases [3,4], intracellular kinases [4-7], and histone methylation [8]. These sensors have been shown to be useful in *in vitro *assays, as well as allow the study of signaling events in a single cell, in real time [4].

The Ras/ERK cascade controls differentiation and proliferation in many different cell types and organisms. In this signal transduction pathway, activated Ras (Ras-GTP) binds directly to Raf-1 and recruits it to the membrane where Raf becomes activated. Raf then phosphorylates and activates Mek-1 and Mek-2 (the MAPK kinase), which in turn phosphorylate the MAPKs ERK-1 and ERK-2. Activated ERKs translocate to the nucleus and directly phosphorylate transcriptional regulatory proteins (including members of the Ets family of transcription factors, and the bZIP factors Fos and Jun) (reviewed in [9]). Kinetics and *in vivo *dynamics of ERK MAP kinase activity are not completely understood. Traditionally, the level of ERK activation has been thought to be relative to the strength of the upstream signal. Some data suggest, however, that ERK activation is "switch-like," requiring a threshold of activation, above which all ERK molecules within the cell become activated [10]. Computational models of MAPK signaling mechanisms can fit both possibilities [11,12]. Therefore, the development of new tools to study these dynamics is essential to determine the biochemical nature of ERK signaling.

In this manuscript, we describe the development of several FRET-based sensors of MAP kinase activity which we have called ERK Activity Sensors (EAS). Peptide linker sequences taken from the Ets family transcription family members Ets1 and Elk1, both ERK targets, were fused between cyan and yellow variants of EGFP (ECFP and EYFP, respectively). Several of our constructs respond to phosphorylation by activated ERK with changes in FRET, and at least one of them, EAS-3, is specific for active Extracellular Regulated Kinase (ERK) as opposed to two other MAP kinases, p38 and SAPK/JNK. Therefore, EAS-3 is a viable, non-radioactive sensor of ERK MAPK activity *in vitro*.

## Results and discussion

### Design and model of EAS

**Figure 1:**
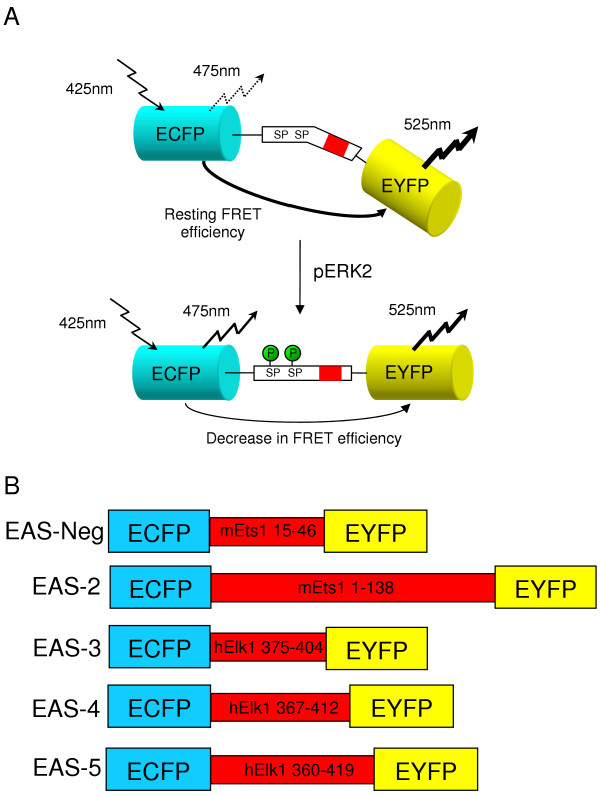
**A model for EAS activation and construct design**. (A) Our inferred model indicates that upon pERK2 phosphorylation a conformational change in the linker peptide of EAS decreases the efficiency of FRET. The red area in the linker peptide indicates the relative position of either D-domain (EAS-2) or DEF domain (EAS-3, -4, -5) and the SP motifs denote the consensus phosphorylation sites. (B) The gene constructs include EAS-Neg and EAS-2, which are derived from mouse Ets1. EAS 3–5 contain peptide linkers derived from human Elk1. The composition of each linker peptide is indicated by the primary sequence positions derived from Ets1 and Elk1, respectively.

EAS was designed to have a short peptide linker sequence sensitive to phosphorylation by ERK fused between ECFP and EYFP. ECFP and EYFP were chosen as a FRET pair because of the good spectral overlap between the emission of ECFP and the excitation of EYFP. We hypothesized that phosphorylation would result in changes in secondary structure of the linker peptide that would alter FRET efficiency for the protein (Figure 1A). Since it was unclear which factors would contribute to forming a suitable FRET sensor, we prepared several different constructs with peptide linker sequences containing ERK phosphorylation sites taken from Ets1 and Elk1 transcription factors, which are physiological substrates of the ERK MAPKs (Figure 1B). EAS-2, derived from mouse Ets1, has a rather large linker peptide, due to the discovery that the MAP Kinase binding site (D-domain) is approximately 90 amino acids downstream of the target serine [13]. The linker sequences for EAS 3–5 were taken from human Elk1, which has been shown to have the DEF domain (FXFP amino-acid motif), a known MAPK binding site, near the C-terminus. These peptides also contain two Serine-Proline (SP) motifs (consensus MAPK phosphorylation sites) corresponding to serines 383 and 389 of Elk1, which have been shown to be targets of MAP kinases. Phosphorylation of these serines has also been shown to be directed specifically by the DEF domain [14]. EAS-Neg is a negative control construct with a short peptide linker derived from Ets1, containing a consensus MAPK phosphorylation site, but no MAPK binding site.

### EAS FRET changes in the presence of active ERK2

**Figure 2:**
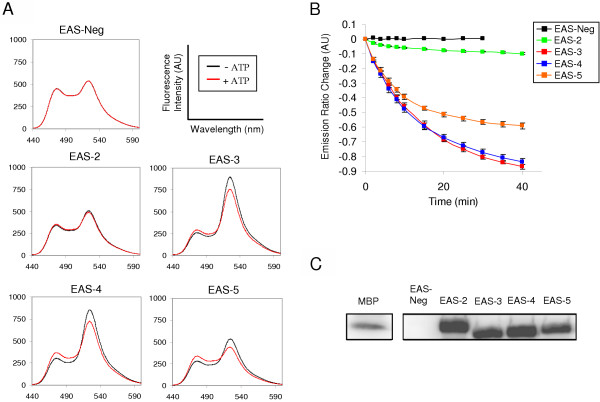
**EAS proteins are targets for ERK2 and exhibit decreased FRETefficiency upon phosphorylation**. (A) Emission spectra indicate the effective FRET change for each EAS protein sensor. Time course fluorimetry with EAS-Neg shows that pERK2 induces no FRET change 30 minutes after ATP addition. All other EAS proteins show varying gains in ECFP emission (475 nm) and losses in EYFP emission (525 nm) 40 minutes after ATP addition. (B) Absolute change in the ratio of EYFP/ECFP emission for EAS-Neg is zero over a 30 minute time course, whereas a decrease in ratio for EAS-3, EAS-4, and EAS-5 is readily detectable at 2 minutes, and continues to decay exponentially over the time course. EAS-2 has a detectable change in emission ratio, but the change is minimal as compared to other EAS proteins. Error analysis was determined from three independent experiments. (C) EAS proteins (EAS-2, -3, -4, and -5) are phosphorylated by pERK2 in the presence of γ-[^32^P]ATP. This confirms that EAS proteins are targets of pERK2 as compared with MBP, a known ERK2 substrate. As expected, EAS-Neg is not phosphorylated by pERK2.

We expected a change in energy transfer between ECFP and EYFP upon incubation with active ERK2 (pERK2) for EAS to be effective as a real time sensor. This would indicate that a phosphorylation event had affected the distance and orientation between the two fluorophores. Each EAS construct was incubated with pERK2 in a fluorimeter (30°C) and readings were taken prior to addition of ATP. Upon the addition of ATP, all EAS constructs, with the exception of EAS-Neg, showed a decrease of energy transfer indicated by an increase in donor (ECFP) emission (475 nm) with a concomitant decrease in acceptor (EYFP) emission (525 nm) after 40 minutes (Figure 2A). Therefore, a decrease in FRET efficiency correlates with phosphorylation of EAS by pERK2. The resultant change in ECFP/EYFP emission ratio is comparable to or exceeds that of similar FRET-based signal transduction sensors [4-6]. Under the same conditions, EAS-Neg showed no change in FRET over the course of 30 minutes, confirming the specificity of ERK2 for EAS 2–5 and the requirement of a phosphorylation event to induce a change in FRET. Figure 2B shows the absolute change in ECFP/EYFP ratio over a 40 minute time course for each construct. EAS-3 and EAS-4 show the greatest absolute decrease in EYFP/ECFP emission ratio, with EAS-5 having an intermediate change in emission ratio. EAS-2 showed very little change in FRET. Phosphorylation was also confirmed by an *in vitro *kinase ^32^P phosphorylation assay using recombinant pERK2 (Figure 2C). EAS 2–5 sensors were efficiently phosphorylated as compared to the non-specific control, EAS-Neg, and Myelin Basic Protein (MBP), a common target of activated MAPKs for *in vitro *studies. Based on the robust FRET signal change and radioassay results, further studies focused on EAS-3.

### EAS changes in FRET in response to pERK2 require phosphorylation and the ERK binding site

Mutants of EAS-3 were utilized to validate the structural features of EAS essential for decreased FRET efficiency upon incubation with pERK2. We refined EAS-3 by replacing the bulky N-terminal GST-purification tag (Glutathione-S-Transferase) with a Histidine-10 tag on the C-terminus. This reduced the possibility that the large purification tag would interfere with FRET efficiency changes. In addition, we mutated alanine 207 and alanine 487 to lysine. As previously reported, this prevents EGFP dimerization [15]. This latter modification reduced co-purification of truncation products with full-length EAS (data not shown).

**Figure 3:**
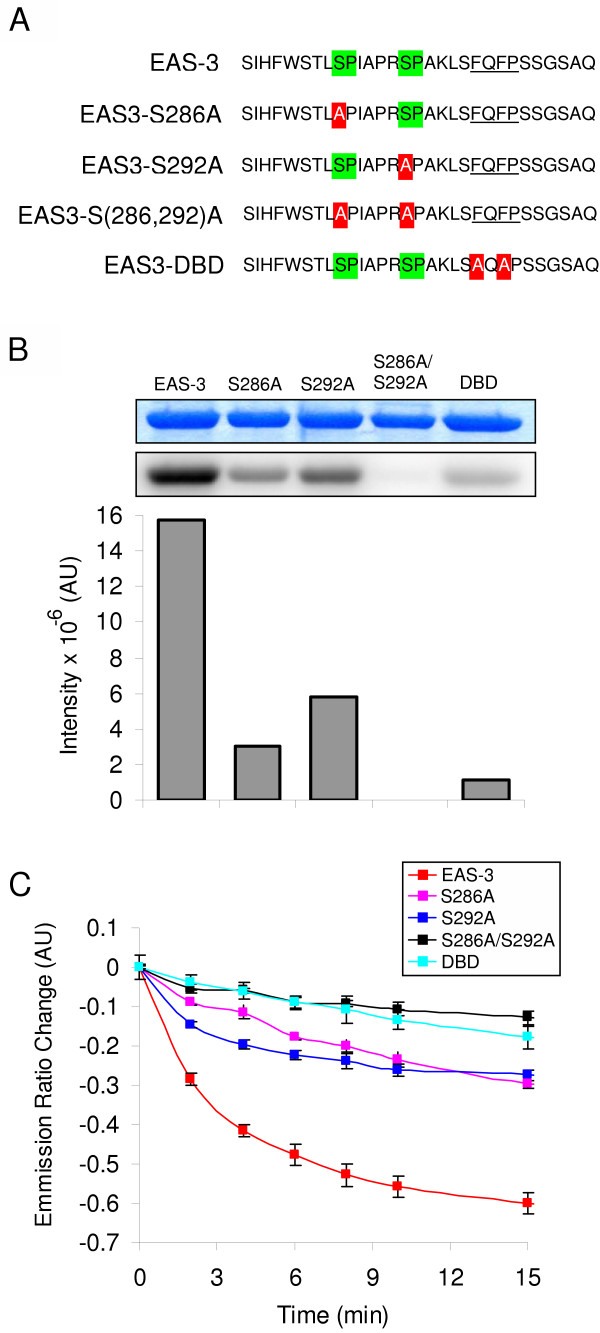
**Mutation of key residues diminishes EAS-3 phosphorylation by pERK2**. (A) Primary sequences of EAS-3 and EAS-3 mutant linker peptides are shown with consensus phosphorylation sites (highlighted in green) and ERK binding sites (underlined). Residue mutations to alanine are highlighted in red. (B) Analysis of mutant EAS-3 proteins by γ-[^32^P]ATP phosphorylation shows complete loss of pERK2 phosphorylation of the S(286,292)A mutant as compared to wild-type EAS-3. Decreased ^32^P-phosphorylation of site-specific mutants also indicates the involvement of both Ser286 and Ser292 for pERK2 activity. Requirement of the ERK binding domain for efficient phosphorylation is evident from decreased phosphorylation of the EAS3-DBD mutant. Band intensities were quantitated by densitometry and are shown graphically. Equal protein loading is shown by Coomassie staining of EAS-3 and EAS-3 mutants and phosphorylation assay was terminated after 15 minutes at 30°C. (C) Fluorimetry data for EAS-3 and EAS-3 mutants reveals that the FRET efficiency change is reduced with various mutations, consistent with the phosphorylation assay in B.

To further characterize EAS-3, we generated different constructs targeting critical residues, and analyzed their ability to serve as substrates for pERK2. Mutants EAS3-S286A, EAS3-S292A, and the double mutant EAS3-S(286,292)A eliminated consensus phosphorylation sites (Figure 2A). The dead binding domain mutant (EAS3-DBD) eliminated the DEF domain by replacing the two key phenylalanines with alanines, creating an AXAP motif (Figure 3A). As Figure 3B demonstrates, all five mutants have decreased phosphorylation compared to the wild type sensor. As expected, the EAS3-S(286,292)A mutant had the lowest level of phosphate incorporation due to absence of both serine-proline consensus phosphorylation sites required by MAP Kinases. A decrease in phosphorylation of EAS-DBD iss also consistent with the requirement of MAPKs to bind targets for efficient phosphorylation (reviewed in [16]). Differences between wild-type EAS-3 and mutants are also reflected in change of FRET efficiency when treated with pERK2 in fluorimeter experiments (Figure 3C). These relative changes in FRET efficiency are consistent with the radioassay data.

### EAS-3 is not phosphorylated by pSAPK or pp38

Distinguishing the activation of different MAP kinases within the cell is essential since each MAPK pathway is activated by multiple mitogens and external environmental factors to varying degrees (reviewed in [17]). Furthermore, there is extensive cross-talk between the different MAP kinase pathways. Detection methods must effectively isolate the signal of the target kinase from other family members to elucidate the contributions of these different pathways to a given cellular process.

**Figure 4:**
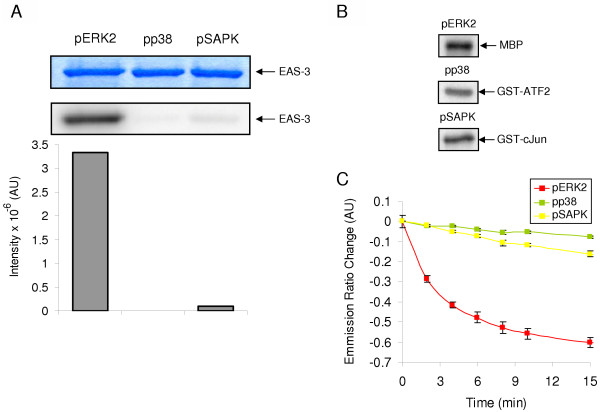
**Determination of ERK2 specificity for EAS-3**. (A) Phosphorylation of EAS-3 is efficient with pERK2, but not pp38 or pSAPK. pp38 and pSAPK incorporates little or no ^32^P into EAS-3 at the same relative specific activity as pERK2. Band intensities were quantitated by densitometry and are shown graphically. Equal protein loading of EAS-3 is shown by Coomassie staining. (B) Specific targets for pERK2 (MBP), pp38 (GST-ATF2), and pSAPK (GST-cJun) are efficiently phosphorylated in the presence of γ-[^32^P]ATP. The amounts of active kinase used in A were based on the relative incorporation of ^32^P into each cognate substrate by the respective kinase. (C) Fluorimetry of EAS-3 with MAPKs shows that the FRET efficiency change is significantly diminished in the presence of pp38 or pSAPK as compared to pERK2. This is consistent with the phosphorylation assay in A.

The target peptide linkers were designed to impart specificity for ERK. Elk1 and Ets1 were chosen because of their seemingly specific interaction with ERK relative to p38 or SAPK/JNK (reviewed in [18]). To determine whether EAS-3 acted as a specific substrate for ERK we performed *in vitro *phosphorylation assays to quantify the ability of pERK2, pp38, and pSAPK/JNK to phosphorylate EAS-3 (Figure 4). As shown in Figure 4A, EAS-3 is an approximately 2000-fold more specific target for pERK2 than pp38, and 50-fold more specific for pERK2 than pSAPK/JNK. Activities of pERK2, pp38 and pSAPK/JNK were confirmed by using known substrates specific for each kinase (Figure 4B). MBP was used in conjuction with pERK2, GST-ATF2 with pp38, and GST-c-Jun with pSAPK/JNK. Specificity of these kinases for EAS-3 was also confirmed by FRET in a fluorimeter (Figure 4C). It is clear that the change in FRET was dramatic when EAS-3 was incubated with pERK2, but showed little or no response when incubated with pp38 or pSAPK/JNK. This is consistent with the results found in 4A. Given the similarity of these kinases, this also suggests that EAS-3 would be specifically acted upon by pERK2 even in the presence of other activated intracellular kinases.

The demonstrated specificity of pERK2 for EAS-3 phosphorylation suggests that this sensor is a candidate for *in vivo *studies of ERK signaling. However, our preliminary experiments in NIH-3T3 cells indicated that EAS-3 was susceptible to intracellular phosphatase activity (data not shown). We surmise that this is due to the absence of a protective phospho-specific binding domain within the EAS-3 construct. Such binding domains have been crucial in the development of other FRET-based signaling sensors of kinase activity [2-4,6,7]. These binding domains, however, are taken from naturally occurring domains that are specific for each target sequence. Unfortunately, no known phospho-specific binding domain exists for Elk1 in the region of Ser383/389. Therefore, we are using a semi-rational approach to develop a phospho-specific binding domain that acts to protect the phosphorylated EAS-3 linker from intracellular phosphatases. This will enable us to adapt the EAS-3 sensor for use in live cells.

## Conclusion

These results show that our novel ERK Activity Sensors provide real-time *in vitro *detection of MAP kinase activity. This method can be applied to studying kinetics of ERK activity in real time, as well as detection of ERK activity in unknown cell lysate fractions. There is also the potential to use these sensors for high-throughput screening of ERK kinase activity with fluorescence plate readers. This method is more direct and convenient to monitor ERK activation *in vitro *than conventional assays that either use radioactivity for detection or rely on indirect detection using phospho-specific antibodies for MAPK targets.

## Methods

### EAS constructs

DNA coding for peptide linkers was amplified by PCR with primers designed with Bgl II and BamHI sites at the 5' and 3' ends, respectively. The products were cloned into pECFP-C1 (Clontech), and EYFP from pEYFP-N1 was subsequently cloned in frame to create EAS constructs. EAS's were cloned into pGEX-2T (Amersham) in frame with GST for expression and purification purposes. These GST-tagged constructs were used for initial fluorimetry experiment of EAS constructs. Other versions of EAS's were constructed using ECFP and EYFP with mutation A(207,487)K, which eliminates dimerization of GFPs, and cloned into pET21a (Novagen) with a Histidine-10 tag. These His10-tagged constructs were used for mutant and specificity experiments. EAS-3 linker mutants were made by Quickchange Site Directed Mutagenesis (Stratagene) to make either single or double point mutation within the peptide linker. For expression in NIH-3T3 cells, EAS's were cloned into the pECFP vector backbone without tags.

### EAS and active kinase expression

BL21(DE3) cells were transformed with EAS constructs and plated on LB agar supplemented with 100 μg/ml ampicillin. Colonies were picked into 4 ml LB-Amp and shaken overnight at 30°C. 250 ml LB was inoculated with starter culture, grown to A_600 _of 0.6, and induced with 0.1 mM IPTG for 18 hours. Cells were spun down, lysed by french press, and cell fragments were spun down at 18,000 rpm in a Beckman JA-20 rotor for 30 min. Proteins were purified from lysates over Ni-NTA Superflow (Qiagen) or Glutathione Sepharose 4B (Amersham).

Activated kinases were purified as described [19]. pERK2 was either purchased (NEB) or purified from bacteria transformed with a plasmid containing both constitutively active MEK1* and His-tagged ERK2. Phosphorylation of ERK2 by MEK1* occurred in bacteria prior to lysis. BL21(DE3) cells were electroporated with MEK1*/ERK2 construct and grown overnight at 37°C on LB-Amp agar plates. Several colonies were picked and incubated in 4 ml of TB supplemented with 100 μg/ml carbenicillin. The starter culture was added to 1L of TB, grown to A_600 _of 0.35, and then induced with 0.25 mM IPTG for 12 hours at 30°C. Cells were harvested and lysed as above, and purified over Ni-NTA Superflow. Activated p38 and SAPK/JNK were purified from bacteria electroporated with two plasmids, one coding for constitutively active MEK kinase 4 (MEKK4*) and another coding for MEK4 and either His-tagged p38 or His-tagged SAPK/JNK. These constructs were grown and purified as above, except with both carbenicillin and kanamycin (50 μg/ml). Protein concentration and buffer exchange performed with Centriplus, Centricon, and Microcon ultrafiltration membranes (Millipore).

### Kinase assays and fluorimetry

Radioactive kinase assays were performed in 25 μl in 12.5 mM MOPS pH 7.5, 12.5 mM β-glycerophosphate pH 7.3, 7.5 mM MgCl_2_, 500 μM NaOrthovanadate, 500 μM NaF, and 9.7 nM DTT. Amounts of kinase and substrate added to reactions are indicated in figure legends. Finally, ATP supplemented with 0.2 nmol [γ-^32^P]ATP (6000 mCi/mmol, Molecular Bioproducts), was added to a final concentration of 1 mM. Reactions were incubated at 30°C for 15 minutes, 8.3 μl of 4× protein sample buffer (SB) were added, and samples were boiled for 5 minutes. Samples were analyzed with 10% or 12% SDS-PAGE (BioRad Mini-Protean II) and transferred to a nitrocellulose membrane. The membrane exposed to a phosphoscreen and scanned on a Storm 860 (Molecular Dynamics). Quantitative densitometry of bands was performed using ImageQuant 5.0 (Molecular Dynamics).

Fluorimetry performed in a Shimadzu Spectrofluorophotometer RF-5301PC. Assays were performed in same buffer as above in a volume of 2.5 ml. The final concentration of EAS constructs was 250 nM and final concentration of pERK2 was 50 nM. The reaction was incubated in fluorimeter and warmed to 30°C. Excitation was set at 425 nm to excite ECFP and avoid excitation of EYFP and readings were taken in 0.2 nm increments. An initial reading was taken prior to ATP addition. To initiate reaction, ATP was added to a final concentration of 1 μM, a spinner was activated for 1 minute, and first reading taken at 2 minutes. EYFP to ECFP ratios were calculated by dividing EYFP peak emission by ECFP peak emission. Peak emissions were defined as an average intensity of EYFP between 524.6–525.4 nm and of ECFP between 474.6–475.4 nm.

## Authors' contributions

HMG performed all experimental work. JAI provided advice, funding and supervision for the work. Both authors have read and approved the final manuscript.
